# Exploring the role of tumor stemness and the potential of stemness-related risk model in the prognosis of intrahepatic cholangiocarcinoma

**DOI:** 10.3389/fgene.2022.1089405

**Published:** 2023-01-12

**Authors:** Yuan Yue, Jie Tao, Dan An, Lei Shi

**Affiliations:** ^1^ Department of Pharmacy, The First Affiliated Hospital of Xi’an Jiaotong University, Xi’an, China; ^2^ Department of Hepatobiliary Surgery, The First Affiliated Hospital of Xi’an Jiaotong University, Xi’an, China

**Keywords:** tumor stemness, mRNAsi, intrahepatic cholangiocarcinoma, molecular subtyping, immune microenvironment, risk model

## Abstract

**Background:** Tumor stem cells (TSCs) have been widely reported to play a critical role in tumor progression and metastasis. We explored the role of tumor stemness in intrahepatic cholangiocarcinoma (iCCA) and established a prognostic risk model related to tumor stemness for prognosis prediction and clinical treatment guidance in iCCA patients.

**Materials and Methods:** The expression profiles of iCCA samples (E-MTAB-6389 and GSE107943 cohorts) were used in the study. One-class logistic regression algorithm calculated the mRNA stemness index (mRNAsi). The mRNAsi-related genes were used as a basis for the identification of mRNAsi-related molecular subtypes through consensus clustering. The immune characteristics and biological pathways of different subtypes were assessed. The mRNAsi-related risk model was constructed with differentially expressed genes (DEGs) between subtypes.

**Results:** The patients with high mRNAsi had longer overall survival than that with low mRNAsi. Two subtypes were identified with that C2 had higher mRNAsi and better prognosis than C1. Tumor-related pathways such as TGF-β and epithelial-mesenchymal transition (EMT) were activated in C1. C1 had higher enrichment of cancer-associated fibroblasts and tumor-associated macrophages, as well as higher immune response and angiogenesis score than C2. We screened a total 98 prognostic DEGs between C1 and C2. Based on the prognostic DEGs, we constructed a risk model containing three genes (*ANO1*, *CD109*, and *CTNND2*) that could divide iCCA samples into high- and low-risk groups. The two groups had distinct prognosis and immune characteristics. Notably, the risk score was negatively associated with mRNAsi (R = −0.53). High-risk group had higher enrichment score of T cell inflamed GEP, INF-γ, and cytolytic activity, and lower score of estimated IC50 of 5-fluorouracil and cisplatin than low-risk group.

**Conclusions:** This study clarified the important role of tumor stemness in iCCA and developed an mRNAsi-related risk model for predicting the prognosis and supporting the clinical treatment in iCCA patients. The three genes (*ANO1*, *CD109*, and *CTNND2*) may serve as potential targets for iCCA treatment.

## Introduction

Tumor stem cells (TSCs) are the cells with the properties of stem cells that enable self-renewal and differentiation, which are responsible for the heterogeneity of tumor cells (Friedmann-Morvinski and Verma, 2014). However, TSCs are not always originated from normal tissue stem cells (Visvader, 2011). The differentiated phenotype of cells was lost during tumor progression, but replaced by the progenitor-like and stem cell-like features, and they are redefined as TSCs. The different status of TSC differentiation in tumor results in intratumoral and intertumoral heterogeneity, and thus shapes the phenotypic heterogeneity. Multiple evidences have demonstrated that TSCs contribute an important role in tumor cell migration, progression, poor prognosis, and the resistance to clinical therapy in different tumors (Shibue and Weinberg, 2017; O'Conor et al., 2018; Pirozzi et al., 2013; Mohanta et al., 2017). Therefore, the classification of different subtypes according to TSC status (tumor stemness) is a viable strategy to identify different prognosis and determine the sensitivity to clinical therapy.

In the majority of solid tumors, the proportion of TSCs less than 3% in whole tumor mass. Surprising, in cholangiocarcinoma (CCA), over 30% of TSCs are existed (Cardinale et al., 2015), suggesting that TSCs contribute a critical role in CCA. CCA is classified into three anatomic subtypes according to the primary, including intrahepatic CCA (iCCA), perihilar CCA (pCCA) or distal CCA (dCCA) (Blechacz et al., 2011). The global age-standardized mortality rates for iCCA increased in the past decades (1-2 per 100,000 in most countries) (Bertuccio et al., 2019). The survival of iCCA patients with lymph node metastasis is poor and benefit little from surgical resection (Kizy et al., 2019). Targeted therapy based on specific gene mutations shows a promising efficiency in some iCCA patients. For example, iCCA patients with isocitrate dehydrogenase (IDH) one mutations have an improved survival after receiving IDH1 inhibitors (ivosidenib) (Hazard ratio, HR = 0.37) in a phase III randomized controlled trial (Abou-Alfa et al., 2020). However, many iCCA patients have no specific gene mutations of IDH1 or fibroblast growth factor receptor (FGFR). Immunotherapy such as immune checkpoint blockade (ICB) has been examined to have a positive efficiency in lines of clinical trials in various tumors. Nivolumab, a programmed cell death protein 1 (PD-1) inhibitor, was administrated in advanced refractory biliary tract cancer and 22% CCA patients showed an objective response (Kim et al., 2020). Identification of CCA subtype with different sensitivity to immunotherapy is essential in the effort to improve the efficiency and outcomes of clinical therapy.

The crosstalk between TSCs and immune microenvironment has been illustrated to affect the efficiency of chemotherapy. Cancer-associated fibroblasts (CAFs) and tumor-associated macrophages (TAMs) are involved in the TSC-induced tumorigenesis and drug resistance through releasing downstream factors (Zhang et al., 2015; Valenti et al., 2017; Ren et al., 2018; Aramini et al., 2021). Malta et al. (2018) dig out transcriptomic (mRNAsi) and epigenetic (mDNAsi) feature sets using used a one-class logistic regression (OCLR) machine-learning algorithm in pan-cancer, and revealed a relationship between immune microenvironment and tumor stemness. Therefore, this study sought to identify tumor stemness-related molecular subtypes and develop an mRNAsi-based risk model. We revealed an association of tumor stemness with prognosis, immune infiltration, and the response to immunotherapy and chemotherapeutic drugs. Negative correlation was found between risk score and mRNAsi. The mRNAsi-based risk model was effective to distinguish the risk of each iCCA patient and manifested a favorable performance in predicting the prognosis of iCCA patients. Especially, the risk model was potential to indicate different response of iCCA patients to immunotherapy and chemotherapy.

## Materials and methods

### Acquisition and preprocessing of iCCA data

E-MTAB-6389 cohort containing microarray data of iCCA samples was obtained from the European Bioinformatics Institute (EBI) webpage (https://www.ebi.ac.uk/arrayexpress/experiments/E-MTAB-6389/). GSE107943 (Ahn et al., 2019) cohort containing gene expression data of iCCA samples was downloaded from Gene Expression Omnibus (GEO) database (https://www.ncbi.nlm.nih.gov/geo/query/acc.cgi?acc=GSE107943). E-MTAB-6389 cohort was used as the training cohort and GSE107943 was determined as the validation cohort.

For E-MTAB-6389 cohort, samples without survival information were excluded. Probes were transferred to gene symbol according to annotation information. The probes matching to multiple genes were removed. The averaged gene expression level was selected when one gene matched multiple probes. After preprocessing, a total of 76 samples were included for analysis.

For GSE107943 cohort, samples without survival information were removed. Fragments per kilobase million (FPKM) format was transferred to transcripts per million (TPM) format. We transformed Ensembl ID into gene symbol. When one gene had multiple gene symbols, we selected the averaged expression. After preprocessing, a total of 30 samples were included for analysis.

### Evaluation of tumor stemness

According to the stemness index model trained from the Progenitor Cell Biology Consortium database, tumor stemness was calculated by one-class logistic regression (OCLR) algorithm (Malta et al., 2018; Wang et al., 2021). Gelnet (v1.2.1) R package was applied to analyze the mRNA stemness index (mRNAsi) of stem cells. Spearman correlation analysis was performed between mRNA expression of tumor samples and the weight vectors of the stemness signature. The stemness index (mRNAsi) reflecting the similarity of tumor cells to stem cells was normalized to range from 0 to 1 through a linear transformation (Malta et al., 2018).

### Identification of mRNAsi-related molecular subtypes

Firstly, mRNAsi-related genes were identified based on the Spearman correlation analysis between mRNAsi and protein-coding genes under criterions of *p* < 0.01 and |correlation coefficient (cor)| > 0.4. To screen mRNAsi-associated genes correlated to cholangiocarcinoma patients’ overall survival, we performed univariate Cox regression analysis. *p* < 0.01 was determined to screen the prognostic mRNAsi-related genes. According to the expression profiles of prognostic mRNAsi-related genes, ConsensusClusterPlus R package (Wilkerson and Hayes, 2010) was applied to conduct unsupervised consensus clustering. PAM algorithm was selected and “1 - Spearman correlation” was used to measuring distance. 500 bootstraps were carried out with each bootstrap including 80% samples of the training cohort. For determining the optimal cluster number k, cumulative distribution function (CDF) curve and consensus matrix were used.

### Analysis of functional pathways

KEGG pathways were acquired from MSigDB (Liberzon et al., 2015). FGSEA R package (Korotkevich et al., 2021) was used to conduct gene set enrichment analysis (GSEA) on KEGG pathways. Pathways showing a false discovery rate (FDR) < 0.05 was significantly enriched. ssGSEA algorithm in GSVA R package (Hänzelmann et al., 2013) was applied to assess the enrichment of KEGG pathways.

### Establishment and validation of an mRNAsi-related risk model

First of all, using limma R package (Ritchie et al., 2015) under conditions of |log2 (fold change)|>log2 (1.5) and *p* < 0.05, differentially expressed genes (DEGs) were identified between different subtypes. ClusterProfiler R package was employed to annotate Gene Ontology (GO) terms and KEGG pathways of DEGs. Then univariate Cox regression was performed on the DEGs to screen those showing a significant correlation with patients’ overall survival (*p* < 0.05). Subsequently, least absolute shrinkage and selection operator (Lasso) regression (Friedman et al., 2010) and stepwise Akaike information criterion (stepAIC) algorithm (Zhang, 2016) were implemented for decreasing prognostic genes number and constructing the optimal risk model. The mRNAsi-related risk model was determined as: risk score = Σ(Exp_i_*β_i_), where i represents genes, Exp represents expression of genes, and β represents Lasso coefficients. Using the median cut-off value of risk score, the samples were divided into two groups of high risk and low risk. Kaplan-Meier survival analysis was conducted to assess the overall survival of two risk groups. Receiver operation characteristic (ROC) curve analysis was used to evaluate the efficiency of the risk model in predicting the overall survival.

### Analysis of immune characteristics

CIBERSORT algorithm was conducted for estimating the proportion of 22 immune cells. ESTIMTAE analysis was used to evaluate immune infiltration and stromal infiltration. 29 immune-related signatures were obtained from a previous study (Bagaev et al., 2021). PROGENy algorithm (Pathway RespOnsive GENes) (Schubert et al., 2018) was used to calculate enrichment score of oncogenic pathways including p53, TGF-β, hypoxia, MAPK, JAK. STAT, NFκB, TNF-α, Trail, EGFR, VEGF, and PI3K.

### Analysis of the sensitivity to immunotherapy and chemotherapeutic drugs

The gene signatures of T cell inflamed gene expression profiles (GEP) (Ayers et al., 2017), Th1/IFN-γ (Danilova et al., 2019), cytolytic activity (Rooney et al., 2015) were obtained from previous research. Eight key immune checkpoints (PDCD1, CTLA4, CD274, TIGIT, PDCD1LG2, LAG3, BTLA, and HAVACR2) were included for predicting the sensitivity to immune checkpoint inhibitors. Pearson correlation analysis was conducted to analyze the correlation of risk score with the immune gene signatures and immune checkpoints using Hmisc R package. The estimated IC50 of three chemotherapeutic drugs (5-fluorouracil, cisplatin, and gemcitabine) was calculated by pRRophetic R package (Geeleher et al., 2014). Based on the drug sensitivity data from Genomics of Drug Sensitivity in Cancer (GDSC) database (Yang et al., 2013) (http://www.cancerrxgene.org), the relation between risk score and drug sensitivity was analyzed by Spearman correlation analysis. Drugs with |Rs| > 0.2 and FDR < 0.05 were considered to have a significant correlation with risk score, where Rs > 0.2 represents drug resistance and Rs < -0.2 represents drug sensitivity.

### Statistical analysis

The bioinformatics analysis was performed with the help of Sangerbox platform (Shen et al., 2022) (http://vip.sangerbox.com/). Log-rank test was performed with Cox regression analysis and survival analysis. Wilcoxon test was performed to test the significance between two groups. Statistical significant was *p* < 0.05. FDR was calculated by Benjamini-Hochberg correction.

## Results

### The relation between mRNAsi and iCCA prognosis

**FIGURE 1:**
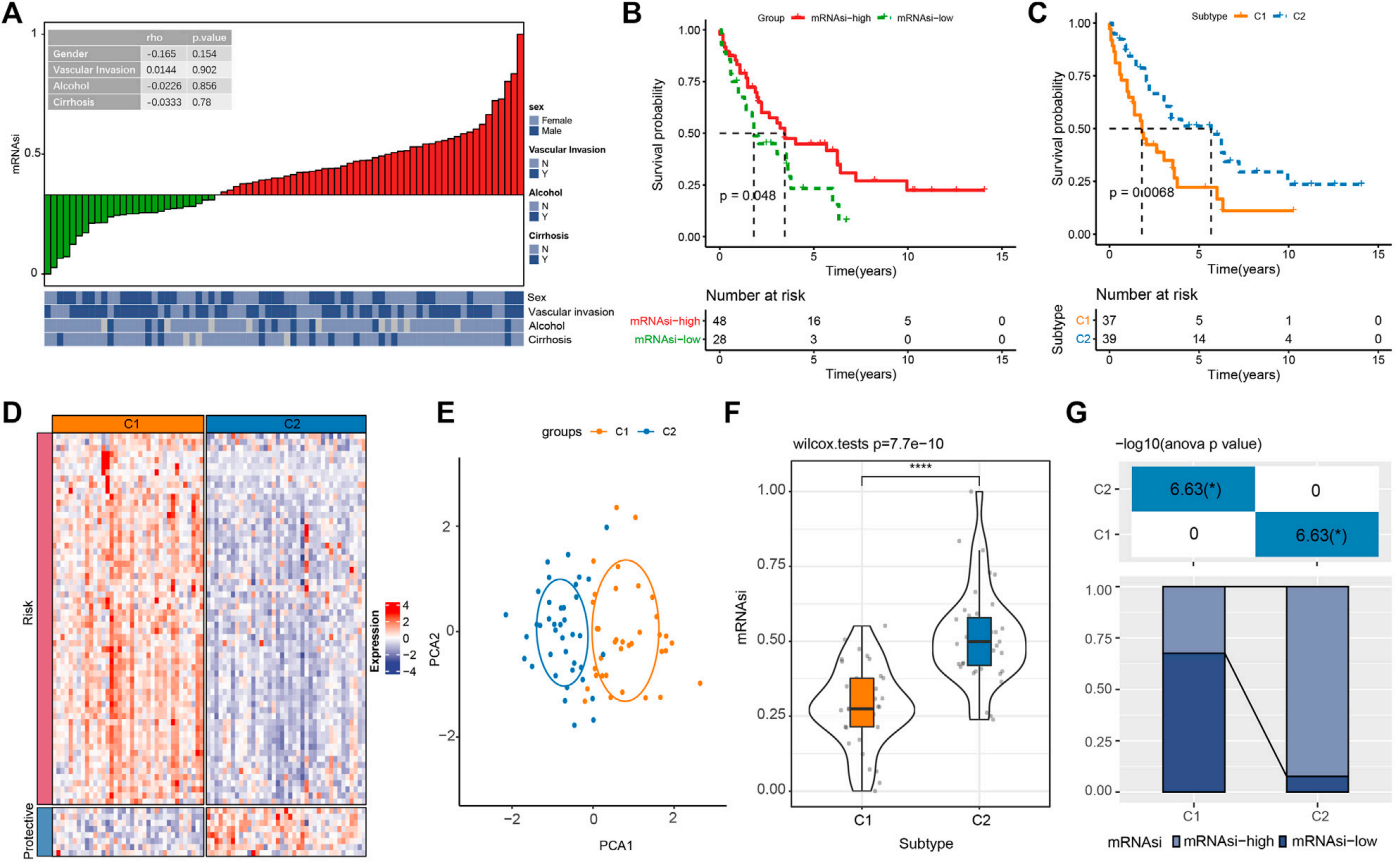
The relation between mRNAsi and the prognosis of cholangiocarcinoma in E-MTAB-6389 cohort. **(A)** Correlation analysis of mRNAsi with clinical features of cholangiocarcinoma patients. **(B)** Kaplan-Meier survival plot of mRNAsi-high and mRNAsi-low groups. **(C)** Kaplan-Meier survival plot of C1 and C2 subtypes. **(D)** Heatmap of the expression (log2TPM) of risk genes and protective genes in C1 and C2. **(E)** PCA plot of C1 and C2. **(F)** Comparison of mRNAsi in C1 and C2. **(G)** The distribution of mRNAsi-high and mRNAsi-low groups in C1 and C2. *p < 0.05, ****p < 0.0001.

We firstly calculated mRNAsi for each iCCA sample in E-MTAB-6389 cohort. On the association of mRNAsi with clinical features (sex, vascular invasion, alcohol, and cirrhosis), no significant correlation was observed (Figure 1A). Then under the optimal cut-off value determined by surv_cutpoint function in survminer R package, cholangiocarcinoma samples were grouped into two mRNAsi groups (mRNAsi-high and mRNAsi-low). Significant difference was detected on the overall survival between mRNAsi-high and mRNAsi-low groups (*p* < 0.05, Figure 1B). To identify the protein-coding genes associated with mRNAsi, we performed Spearman correlation analysis and screened a total of 1794 mRNAsi-related genes (|cor| > 0.4 and *p* < 0.01). Subsequently, we identified a total of 69 prognostic genes within 1794 miRNA-related genes through univariate Cox regression analysis, where 61 risk genes (HR > 1) and 8 protective genes (HR < 1) were included (Supplementary Figure S1A).

To further understand the association of mRNAsi with iCCA prognosis, we applied consensus clustering to identify mRNAsi-associated molecular subtypes based on the 69 prognostic miRNA-related genes. To clustering samples into two subtypes (C1 and C2), cluster number k = 2 was determined (Supplementary Figures S1B–D). Kaplan-Meier survival analysis on the two subtypes showed that C2 had significantly longer overall survival than C1 (*p* < 0.01, Figure 1C). Risk genes were relatively higher expressed and protective gene were relatively lower expression in C1 compared with that in C2 (Figure 1D). In addition, PCA showed a separated distribution of expression profiles of two subtypes (Figure 1E). C2 showed a significantly higher mRNAsi than C1 (Figure 1F), and mRNAsi-high samples also contributed a higher percentage in C2 (Figure 1G), indicating that high mRNAsi was probably a protective factor in iCCA prognosis. Moreover, we found a positive correlation between mRNAsi with CDH1 (epithelial marker) and a negative correlation between mRNAsi and CDH2 (mesenchymal marker) (Supplementary Figure S2), suggesting a negative relation between mRNAsi and cancer metastasis in iCCA. The results were consistent with the previous study (Malta et al., 2018).

### Differential enrichment of biological pathways in two subtypes

**FIGURE 2:**
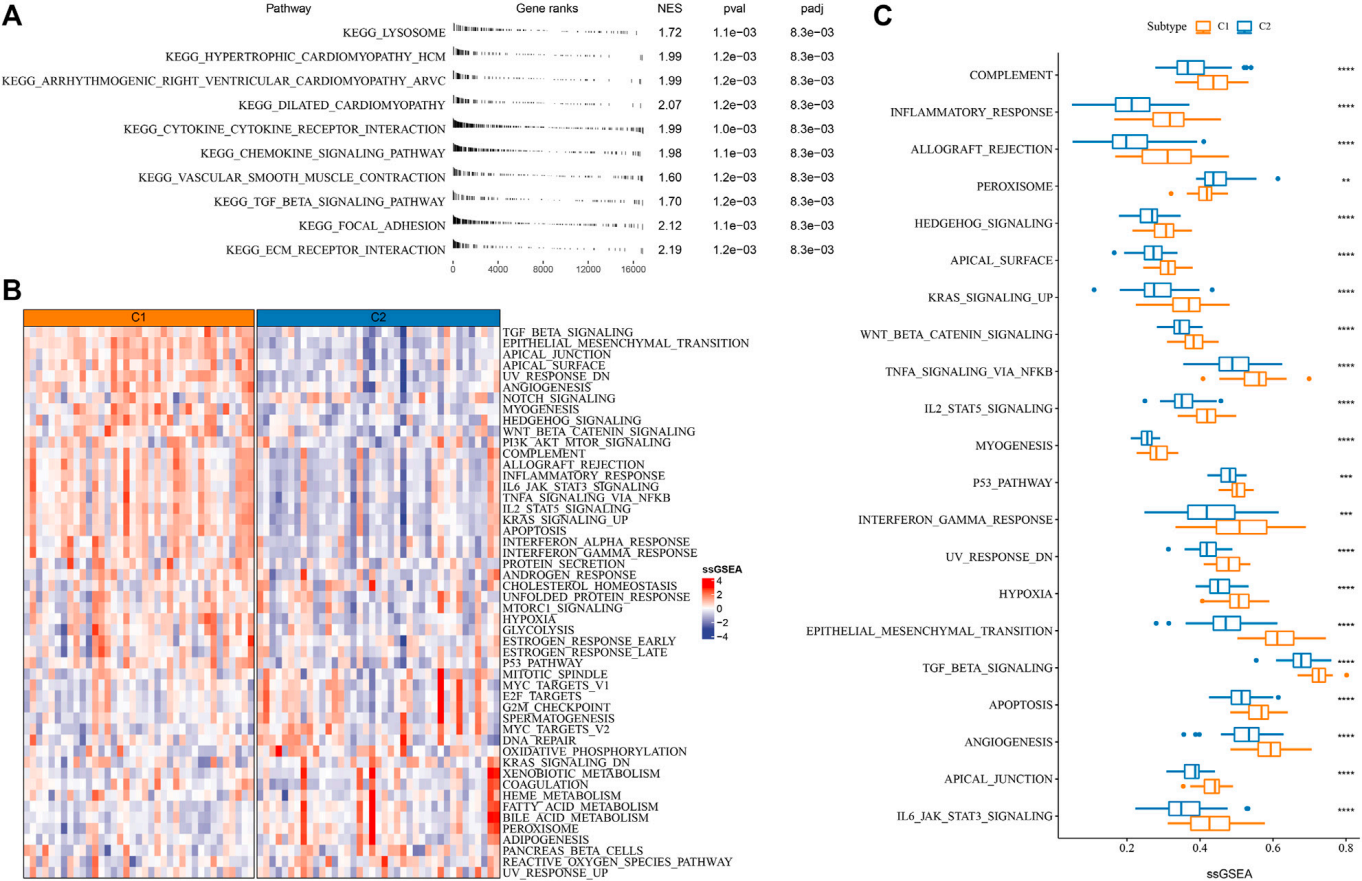
Enrichment of functional pathways in C1 and C2 of E-MTAB-6389 cohort. **(A)** GSEA results displaying significantly enriched KEGG pathways of C1. **(B)** Heatmap of the top 50 enriched pathways in C1 and C2. **(C)** A total of 21 pathways differentially enriched between C1 and C2. Wilcoxon test was conducted. **p < 0.01, ***p < 0.001, ****p < 0.0001.

As mRNAsi was associated with the prognosis of iCCA patients, we attempted to reveal the potential biological pathways involved in tumor stemness. GSEA was performed on all candidate gene sets of KEGG pathways and the significantly enriched pathways in C1 were outputted (FDR < 0.05). In C1 than C2 immune and stromal pathways were more activated, such as cytokine-cytokine receptor interaction, focal adhesion chemokine, and ECM receptor interaction, signaling pathway, (Figure 2A). Moreover, ssGSEA results revealed that tumor-related pathways (for example, epithelial-mesenchymal transition, TGF-β signaling, angiogenesis, Wnt-β signaling, Notch signaling and PI3K-Akt signaling, P53 signaling, hypoxia) and immune-related pathways (for example, IL6-JAK-STAT3 signaling, complement, inflammatory response, interferon response) showed a significantly higher enrichment score in C1 (*p* < 0.001, Figures 2B, C). The above findings suggested a correlation between tumor stemness and immune modulation, and tumor stem cells was involved in the tumor development through activating oncogenic pathways in iCCA.

### The immune characteristics of two subtypes

**FIGURE 3:**
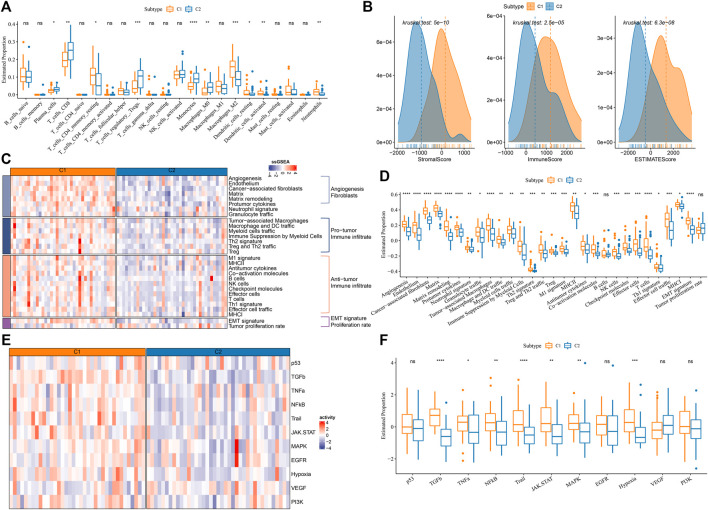
Immune characteristics in C1 and C2 of E-MTAB-6389 cohort. **(A)** The distribution of 22 immune cells in C1 and C2 analyzed by CIBERSORT. Wilcoxon test was conducted. **(B)** ESTIMATE analysis showed the immune score, stromal score and ESTIMATE score of C1 and C2. **(C)** Heat map of 29 immune-related signatures in C1 and C2. **(D)** Box plot showing the ssGSEA score of 29 immune-related signatures in C1 and C2. Wilcoxon test was conducted. **(E)** Heatmap of oncogenic pathways. **(F)** Box plot of oncogenic pathways. Wilcoxon test was conducted. ns, not significant. *p < 0.05, **p < 0.01, ***p < 0.001, ****p < 0.0001.

In the previous section, we demonstrated that C1 and C2 had differential enrichment of immune-related pathways. We next evaluated the immune microenvironment of C1 and C2 by different tools. CIBERSORT analysis on 22 immune cells showed that some immune cells were differentially enriched between two subtypes, such as higher enrichment of CD8 T cells, regulatory T cells, monocytes, M0 macrophages in C2, but higher enrichment of M2 macrophages in C1 (*p* < 0.01, Figure 3A). ESTIMATE results presented that C1 had evidently higher immune infiltration and stromal infiltration than C2 (*p* < 0.0001, Figure 3B). Furthermore, we collected some immune-related gene signatures from a previous study (Bagaev et al., 2021), and calculated their enrichment scores using ssGSEA. As shown in Figures 3C, D, the angiogenesis-related signatures, cancer-associated fibroblasts (CAFs), pro-tumor signatures and epithelial-mesenchymal transition (EMT) signature were relatively activated in C1; at the same time, anti-tumor signatures were also more enriched in C1 than that in C2. In 11 oncogenic pathways, 7 of them were more significantly activated in C1 than that in C2 (*p* < 0.05, Figures 3E, F). Although C1 had an anti-tumor immune microenvironment, pro-tumor activity led it to a more progressive outcome than C2.

**FIGURE 4:**
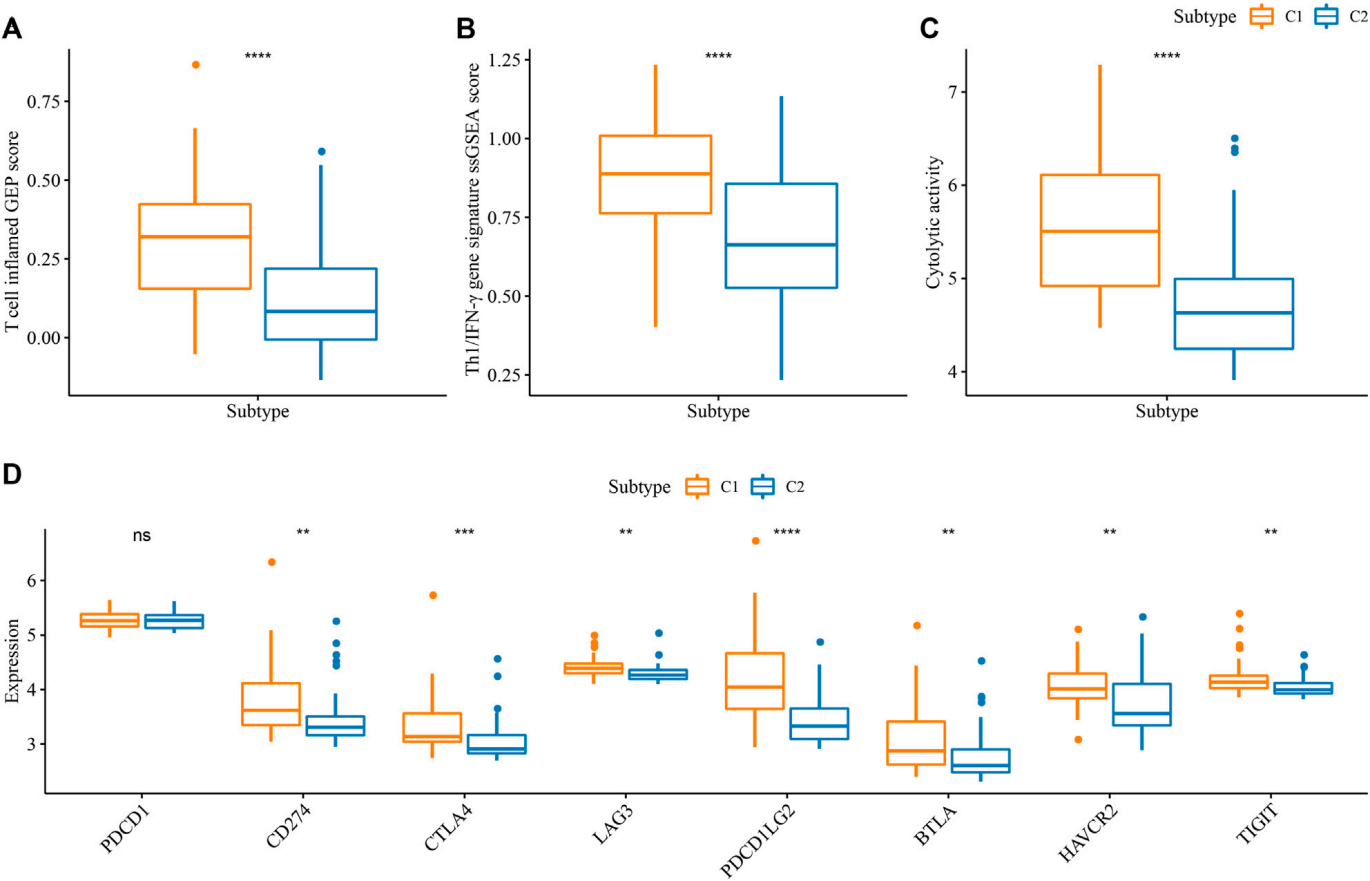
Prediction of the response to immunotherapy in E-MTAB-6389 cohort. **(A–C)** Comparison of ssGSEA score of T cell inflamed GEP, Th1/IFN-γ signature, and cytolytic activity in C1 and C2. **(D)** The expression of key immune checkpoints in C1 and C2. Wilcoxon test was conducted. ns, not significant. **p < 0.01, ***p < 0.001, ****p < 0.0001.

In addition, we examined the potential immune response of two subtypes to immunotherapy. It has been reported that T cell inflamed GEP score and Th1/IFN-γ are positively associated with anti-tumor response in immunotherapy (Ribas and Hu-Lieskovan, 2016; Ott et al., 2019). C1 displayed higher scores of T cell inflamed GEP, Th1/IFN-γ, as well as cytolytic activity than C2 (*p* < 0.0001, Figures 4A–C), indicating that C1 was predicted to be more responsive in immunotherapy. Immune checkpoint inhibitors (ICIs) such as PD-1 and PD-L1 are important factors in immune checkpoint blockade therapy. High PD-1/PD-L1 expression has been demonstrated to associate with high sensitivity to ICIs (Patel and Kurzrock, 2015). In the eight key immune checkpoints, we found that their expression levels except for PD-1 (PDCD1) were significantly higher in C1 than that in C2 (*p* < 0.01, Figure 4D). The above observations implied that C1 was more sensitive to immunotherapy than C2.

### Establishment and validation of an mRNAsi-related prognostic model for iCCA

Given that C1 and C2 had distinct immune microenvironment and activated biological pathways, we identified a total of 1746 DEGs (FDR < 0.05, log2FC > 0) between C1 and C2 using limma R package (Supplementary Table S1). Of these DEGs, SERINC1 and MYO9B were previously reported as potential driver genes in liver cancer (Basu et al., 2018). Then, we screened 473 DEGs with 1.5 -fold change, and 401 up-regulated genes and 72 down-regulated genes in C1 were outputted (Supplementary Figures S3A, B). Gene enrichment analysis on these DEGs showed that immune-related GO terms and pathways were annotated in up-regulated genes, which was accordant with the findings in the previous section (Supplementary Figure S3C; Figure 2). In down-regulated genes, metabolism-related pathways and terms were enriched in C2 such as drug metabolism and tyrosine metabolism (Supplementary Figure S3D).

**FIGURE 5:**
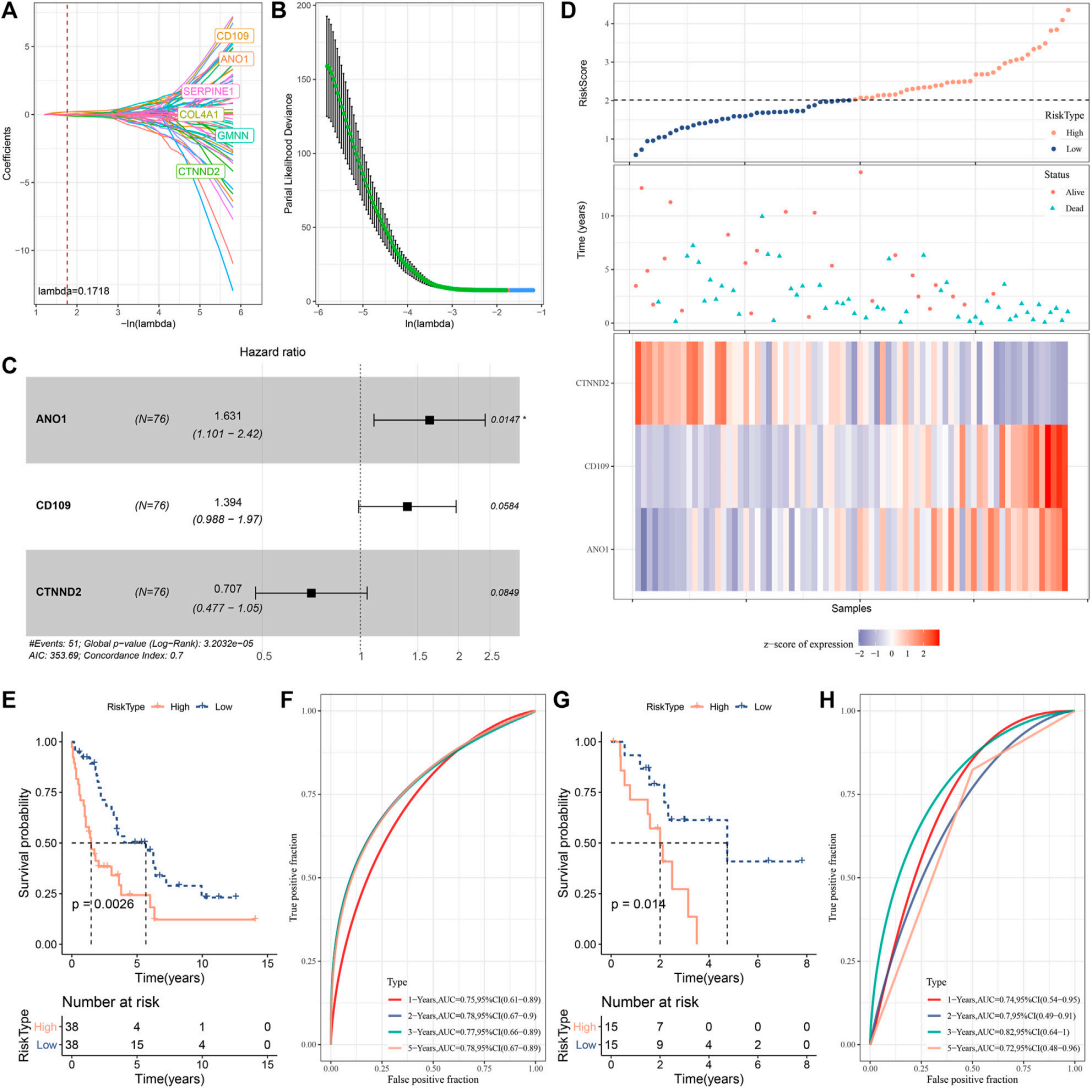
Construction and validation of an mRNAsi-related risk model. **(A, B)** Lasso regression on the 98 prognostic genes. Red dashed line in **(A)** and red dot in **(B)** represents lambda = 0.1718. **(C)** Three prognostic genes were screened by stepAIC. Log-rank test was conducted. **(D)** The risk score, survival status, and the expression of three genes of all samples in E-MTAB-6389 cohort. **(E)** Survival plot of high- and low-risk groups in E-MTAB-6389 cohort. **(F)** ROC curve of the risk model in predicting 1-year, 2-year, 3-year, and 5-year survival in E-MTAB-6389 cohort. **(G, H)** Survival plot and ROC curve of the risk model in GSE107943 cohort. *p < 0.05.

The DEGs were used as a basis to construct a prognostic model in the training cohort (E-MTAB-6389). Univariate Cox regression on the 473 DEGs identified a total of 98 DEGs including 86 risk genes and 12 protective genes significantly associated with overall survival (Supplementary Table S2). Then to decrease the number of prognostic genes for constructing an optimal model, Lasso regression was employed here. When lambda = 0.1718, the model reached the optimal, and six prognostic genes were remained (Figures 5A, B). Furthermore, we applied stepAIC to obtain the sufficient fitting degree with the least number of variables (genes). Finally, three genes were remained including *ANO1*, *CD109*, and *CTNND2* (Figure 5C). The mRNAsi-related prognostic model was defined as: Risk Score = 0.489*ANO1 + 0.332*CD109–0.346*CTNND2.

The risk score was calculated for training cohort samples. The median value of risk score was used to group samples into two groups of high and low risk (Figure 5D). The expression levels of ANO1 and CD109 were relatively higher in high-risk group while CTNND2 was relatively lower expressed compared with low-risk group. From the results of survival analysis, patients with a high risk evidently developed a worse overall survival than those in the low-risk group (*p* < 0.01, Figure 5E). ROC curve analysis illustrated that the model had a high efficiency in predicting 1-year, 2-year, 3-year, and 5-year overall survival with AUC of 0.75, 0.78, 0.77, and 0.78 respectively (Figure 5F). We verified the risk model in the validation cohort (GSE107943), and observed the similar results (Figures 5G, H). We also compared the risk score of mRNAsi-low and mRNAsi-high, as well as C1 and C2. The mRNAsi-low group and C1 subtype exhibited a higher risk score than the mRNAsi-high group and C2 subtype (*p* < 0.0001, Supplementary Figures S4A, B). The high-risk samples contributed to a high percentage in C1 subtype and mRNAsi-low group (Supplementary Figure S4), which was consistent with their prognosis. The mRNAsi-related risk model also showed a favorable performance distinguishing high-risk samples in different mRNAsi groups and subtypes (Supplementary Figure S4D). It could be concluded that the mRNAsi-related risk model was robust in predicting the prognosis of iCCA patients.

### The relation of risk score with biological pathways and immune microenvironment

**FIGURE 6:**
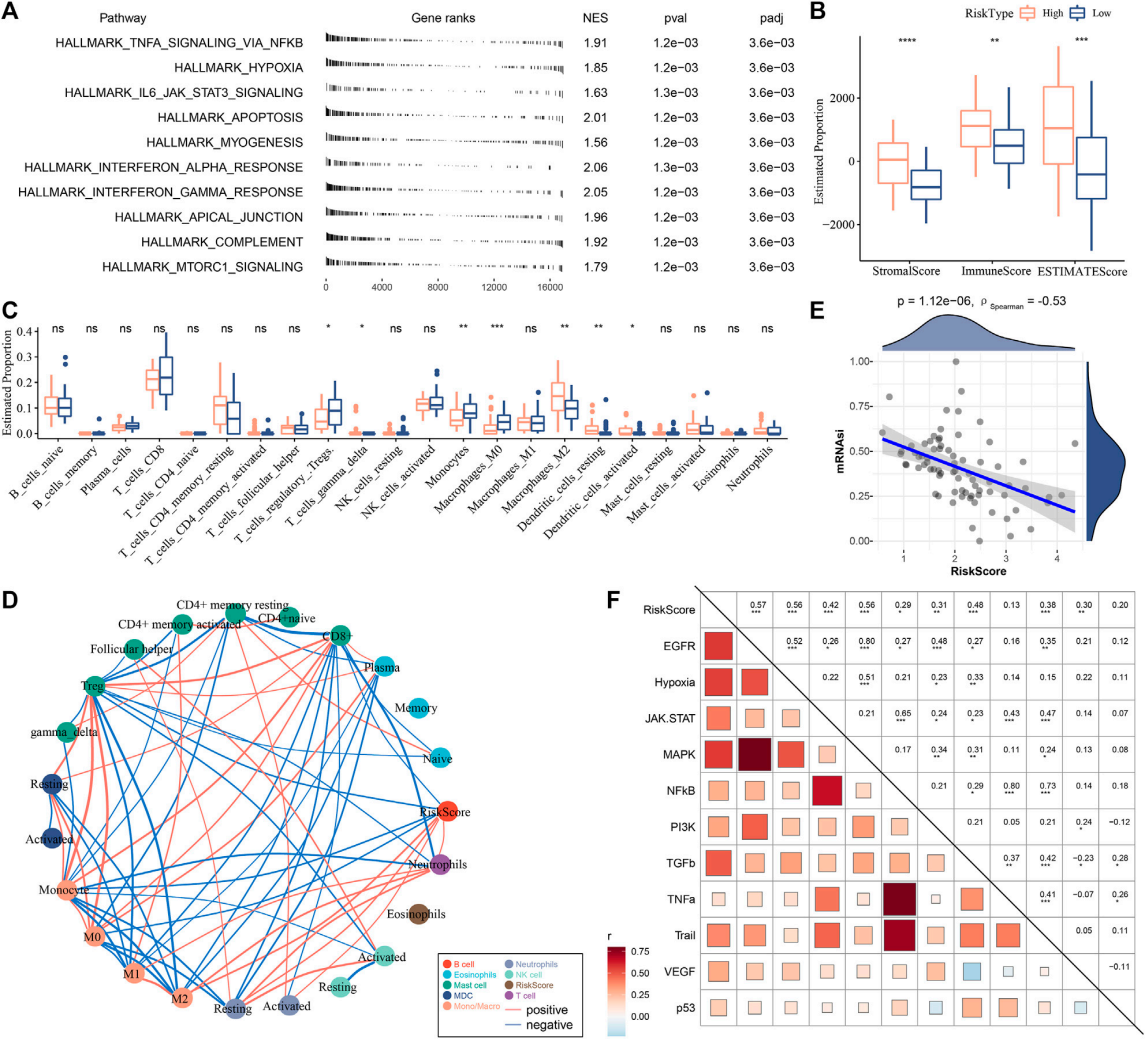
Analysis of biological pathways and immune microenvironment in two risk groups in E-MTAB-6389 cohort. **(A)** GSEA results of significantly enriched pathways in high-risk group. **(B)** ESTIMATE analysis showed immune score, stromal score and ESTIMATE score of two risk groups. Wilcoxon test was conducted. **(C)** CIBERSORT analysis showed the enrichment of 22 immune cells in two risk groups. Wilcoxon test was conducted. **(D)** Pearson correlation analysis of risk score with immune cells. Red and blue lines indicate positive and negative correlation respectively. **(E)** Spearman correlation analysis between mRNAsi and risk score. **(F)** Pearson correlation analysis of risk score with oncogenic pathways. ns, not significant. *p < 0.05, **p < 0.01, ***p < 0.001, ****p < 0.0001.

We assessed the biological pathways of two risk groups using GSEA. Immune-related pathways such as interferon-gamma response, interferon-alpha response, IL6-JAK-STAT3 signaling, and complement were evidently enriched in high-risk group (Figure 6A), suggesting that immune response was more activated in patients with a high risk than those in low-risk group. We examined the immune infiltration of two risk groups, and found that high-risk group had significantly greater immune infiltration and stromal infiltration (Figure 6B). Patients showing a low risk had higher enrichment of regulatory T cells and M0 macrophages, monocytes, but had lower enrichment of M2 macrophages than high-risk group (Figure 6C). Moreover, the relationship of risk score with the infiltration of different immune cells was evaluated. The risk score was positively correlated with M2 macrophages, and was negatively correlated with CD8 T cells, regulatory T cells, monocytes and M0 macrophages (Figure 6D). The immune characteristics in high-risk group were consistent with that in C1 (Figures 3A, B). Therefore, we speculated that there was an association of the risk score with mRNAsi. Not surprisingly, a significantly negative correlation was revealed by Spearman correlation analysis between mRNAsi and the risk score (*p* < 0.0001, R = −0.53, Figure 6E). The result indicated that high-risk group had a lower mRNAsi than low-risk group, which accounted for the consistence of immune characteristics between risk groups and subtypes. In addition, the risk score was positively correlated with tumor-related pathways such as EGFR (R = 0.57), hypoxia (R = 0.56), MAPK (R = 0.56), and TGF-β (R = 0.38) (Figure 6F), and immunosuppressive features such as angiogenesis (R = 0.33), CAFs (R = 0.50), and TAMs (R = 0.55) (Supplementary Figure S5), which was similar to the previous results (Figure 3F).

### Different responses of two risk groups to immunotherapy and chemotherapy

**FIGURE 7:**
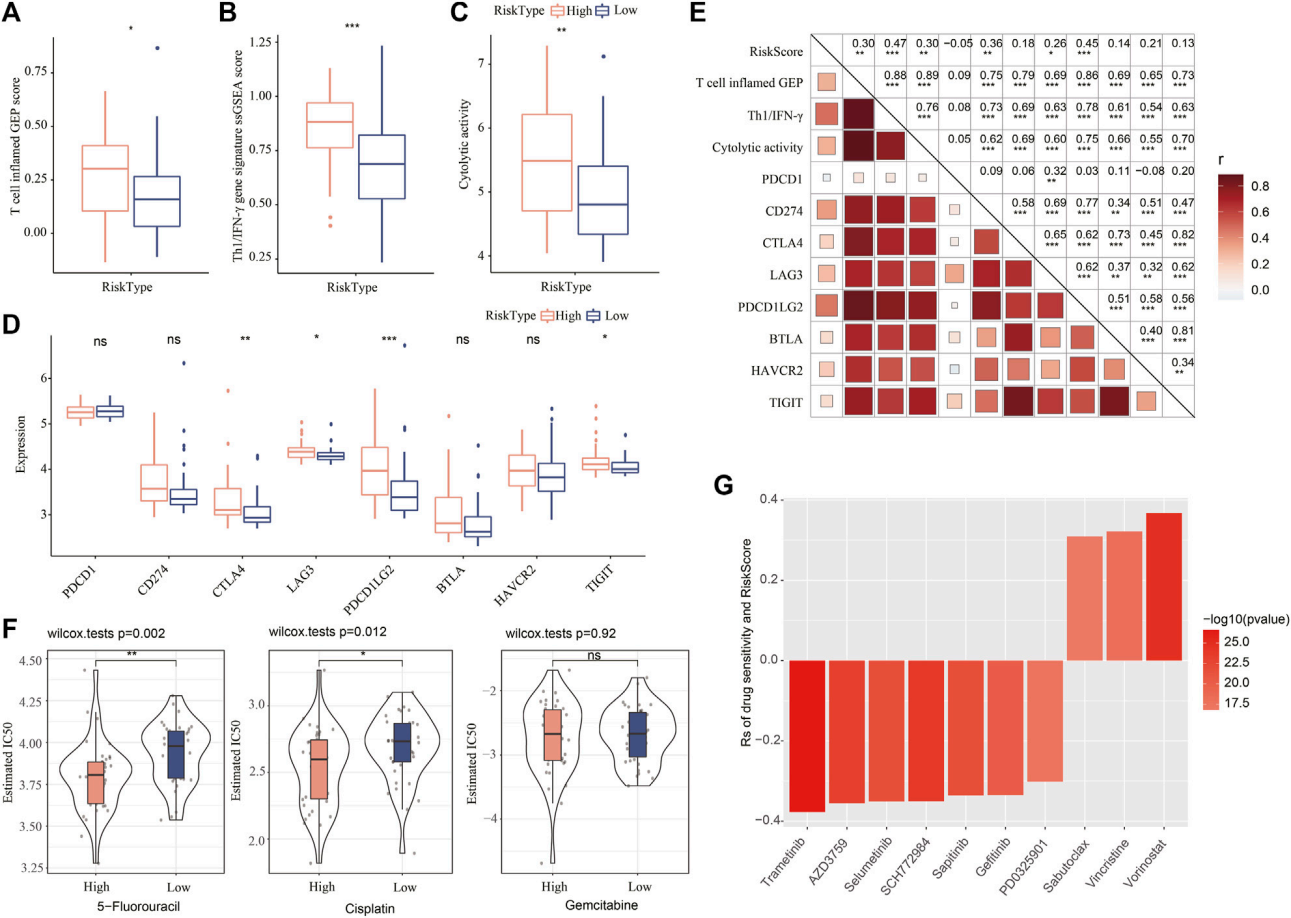
Validating the value of the risk model in predicting the response to clinical therapy. **(A–C)** The ssGSEA score of T cell inflamed GEP, Th1/IFN-γ, and cytolytic activity in two risk groups. Wilcoxon test was performed. **(D)** The expression of key immune checkpoints in two risk groups. Wilcoxon test was performed. **(E)** Pearson correlation analysis of risk score with immune signatures and immune checkpoints. **(F)** The estimated IC50 of 5-fluorouracil, cisplatin, and gemcitabine in two risk groups. **(G)** Spearman correlation analysis between drug sensitivity and risk score. Rs indicates correlation coefficient.

The mRNAsi-related risk model was verified to be effective in predicting the prognosis of iCCA in different cohorts, and two risk groups showed differential mRNAsi, immune microenvironment, and activation of biological pathways. We further examined the value of the risk model in guiding clinical therapies. High-risk group was suggested to have a higher sensitivity to immunotherapy than low-risk group according to the higher score of T cell inflamed GEP, Th1/IFN-γ, and cytolytic activity in high-risk group (*p* < 0.05, Figures 7A–C). Moreover, the expression level of key immune checkpoints was also lower in low-risk group (Figure 7D). Correlation analysis of the risk score with the above indicators of immunotherapy displayed that the risk score showed a positive correlation with T cell inflamed GEP (R = 0.30, *p* < 0.01), Th1/IFN-γ (R = 0.47, *p* < 0.001), cytolytic activity (R = 0.30, *p* < 0.01), CD274 (PD-L1) (R = 0.36, *p* < 0.01), LAG3 (R = 0.26, *p* < 0.05), and PDCD1LG2 (R = 0.45) (Figure 7E, *p* < 0.0001).

In the response to chemotherapeutic drugs, we used estimated IC50 to predict the response of two risk groups to 5-fluorouracil, cisplatin, and gemcitabine. High-risk group was shown to have lower estimated IC50 of 5-fluorouracil and cisplatin than low-risk group, indicating that high-risk group was more sensitive to the two drugs. In addition, we obtained the data of drug sensitivity of about 190 drugs in 1000 cancer cell lines from GDSC, and analyzed the correlation between the risk score and the sensitivity to these drugs. As a result, 10 drugs were found to be significantly correlated with the risk score, where 7 drugs showed drug sensitivity relating to risk score (Rs < −0.2) and 3 drugs showed drug resistance relating to risk score (Rs > 0.2, Figure 7F). The results suggested that the 7 drugs (trametinib, AZD3759, selumetinib, SCH772984, sapitinib, gefitinib, and PD0325901) were predicted to the potential therapeutic drugs in iCCA. The mRNAsi-related risk model was potential to estimate the sensitivity to immunotherapy or chemotherapeutic drugs.

## Discussion

Tumor stemness has been uncovered to have an effect in tumorigenesis, tumor progression and metastasis. This study used the expression data of iCCA for evaluating tumor stemness at a transcriptional level (mRNAsi) of iCCA patients. High-mRNAsi and low-mRNAsi groups showed a significantly different overall survival. The patients with high mRNAsi had longer overall survival than that with low mRNAsi, indicating that tumor stemness was involved in the iCCA development. To further reveal the link of tumor stemness with iCCA prognosis, we identified mRNAsi-related molecular subtypes based on the expression data of mRNAsi-related prognostic genes. Two subtypes were identified and C1 and C2 subtypes showed distinct expression patterns. In addition, C2 had a higher mRNAsi level and more favorable prognosis than C1. We preliminarily confirmed the association between mRNAsi and iCCA prognosis.

To clarify the potential mechanism of tumor stemness contributing to iCCA development, we assessed the functional pathways of C1 and C2. Tumor-related pathways especially TGF-β signaling and EMT, Notch signaling, and Wnt signaling were more enriched in C1 than that in C2. EMT is a biological process enabling epithelial cells to acquire mesenchymal phenotypes, which can be triggered by TGF-β (David et al., 2016). Compelling evidence has shown that tumor cells have activated EMT process that allows tumor cells gaining invasive features (Wilson et al., 2020). In EMT process, tumor cells acquired stemness that can increase motility and promote metastasis (Dongre and Weinberg, 2019). Although the specific transition states of EMT inducing stemness have not been fully defined, EMT in promoting stemness is supported by the involvement of Wnt signaling (Basu et al., 2018), Notch signaling (Fender et al., 2015), Mitofusin signaling (Wu et al., 2019), and Hedgehog signaling pathways (Guen et al., 2017).

In addition to EMT-related pathways, immune-related pathways such as angiogenesis, complement, PI3K-Akt-mTOR signaling, IL6-Jak-Stat3 signaling, inflammatory response, interferon response, IL2-Stat5 signaling were also more enriched in C2 compared with C1. Not surprisingly, C1 had higher immune response than C2, which showed as higher immune infiltration, T cell inflamed GEP score, and cytolytic activity. However, it seemed controversial with the outcome that C1 had a worse prognosis. At the same time, C1 also exhibited an immunosuppressive environment that CAFs and TAMs were evidently accumulated. Multiple inflammatory modulators promotes CAF activation, such as interleukin-1 (IL-1) acting through NF-κB and IL-6 acting on STAT transcription factors (Erez et al., 2010; Sanz-Moreno et al., 2011). In our results, NF-κB and JAK-STAT signaling were more activated in C1 compared with C2. High TAM infiltration is associated with poor prognosis in solid tumors, as well as the association with angiogenesis, migration, and the resistance to chemotherapy and radiotherapy (Chen et al., 2019). Moreover, we found that C1 had higher expression levels of key immune checkpoints such as PDL1, CTLA4, and LAG3, which was also responsible for the immunosuppressive environment. From the above analysis, we considered that the activation of EMT, oncogenic pathways, and the enrichment of immunosuppressive cells were the main contributors for the poor overall survival of C1.

Given that two mRNAsi-related subtypes had significantly different molecular features, we then screened a group of prognostic DEGs that may be involved in iCCA progression. By using Lasso and stepAIC algorithm, we constructing a prognostic risk model containing three genes (ANO1, CD109, and CTNND2). The risk score was calculated for each iCCA sample and they were divided into high-risk and low-risk groups according to the median risk score. In both training and validation cohorts, high-risk group had a worse overall survival than low-risk group, and the risk model showed a favorable performance in predicting 1-year, 3-year, and 5-year survival with AUC over than 0.70. The expression levels of three genes were associated with the risk score, where ANO1 and CD109 were highly expressed in high-risk group and CTNND2 was highly expressed in low-risk group.

Notably, we discovered that risk score was negatively correlated with mRNAsi (R = −0.53), which showed a consistence with the finding that low mRNAsi was associated with poor prognosis. The results indicated that the three genes in the risk model were importantly involved in the regulation of tumor stemness. ANO1 was found to be a risk factor in many cancer types (HR = 1.52, 95% CI: 1.19-1.92), and was suggested to be a prognostic factor (Zhang et al., 2021). Kim et al. uncovered that ANO1 knockdown could increase the survival and inhibit local invasion of glioblastoma stem cells (GSCs) in mouse model, indicating that ANO1 was important in the maintenance of stemness (Kim et al., 2021). In human lung adenocarcinoma cell lines, CD109 overexpression was associated with the ability of migration and metastasis by activating the Jak-Stat3 signaling (Chuang et al., 2017). Actually, Jak-Stat3 signaling was more activated in high-risk group than that in low-risk group. Previous research revealed that CD109 promoted EMT process and stemness in lung adenocarcinoma, and CD109 was considered as a potential therapeutic target (Lee et al., 2020). CTNND2 (δ-catenin) was suggested as a potential cancer biomarker and was associated with the expression of markers of cancer stem cells in lung adenocarcinoma (Lu et al., 2014; Huang et al., 2018). However, the roles of these three genes in CCA have not been revealed in the previous research. Our study only provided a direction for the further analysis of their function in tumor stemness in CCA, and further experiments are needed to verify the roles of three genes in the future work.

We characterized the biological features of high- and low-risk groups, and the results were consistent with that in subtype analysis. High-risk group had significantly higher immune infiltration and more activated immune response than low-risk group. Simultaneously, immunosuppressive environment was more enriched in high-risk group, such as high enrichment of angiogenesis, CAFs, and TAMs, as well as high expression of immune checkpoints, which contributed for the unfavorable outcome of high-risk group. Nevertheless, the prediction of sensitivity to immunotherapy and chemotherapy revealed that high-risk group was more sensitive to immune checkpoint inhibitors and chemotherapeutic drugs such as 5-fluorouracil and cisplatin. The results laid a foundation for the predictive value of the mRNAsi-related risk model in clinical treatment for iCCA patients.

## Conclusion

In conclusion, this study clarified the relation of tumor stemness with prognosis and immune microenvironment in iCCA patients. In addition, we constructed an mRNAsi-related risk model that was effective and stable to predict the overall survival of iCCA patients. Importantly, the risk model showed a potential to predict the sensitivity of iCCA patients to immunotherapy and chemotherapeutic drugs.

## Data Availability

The original contributions presented in the study are included in the article/Supplementary Material, further inquiries can be directed to the corresponding author.
