# Supplementary figures and images for: Exploring the role of tumor stemness and the potential of stemness-related risk model in the prognosis of intrahepatic cholangiocarcinoma
Source: Front Genet. 2023 Jan 12;13:1089405. doi: 10.3389/fgene.2022.1089405 (PMC9877308; doi:10.3389/fgene.2022.1089405)

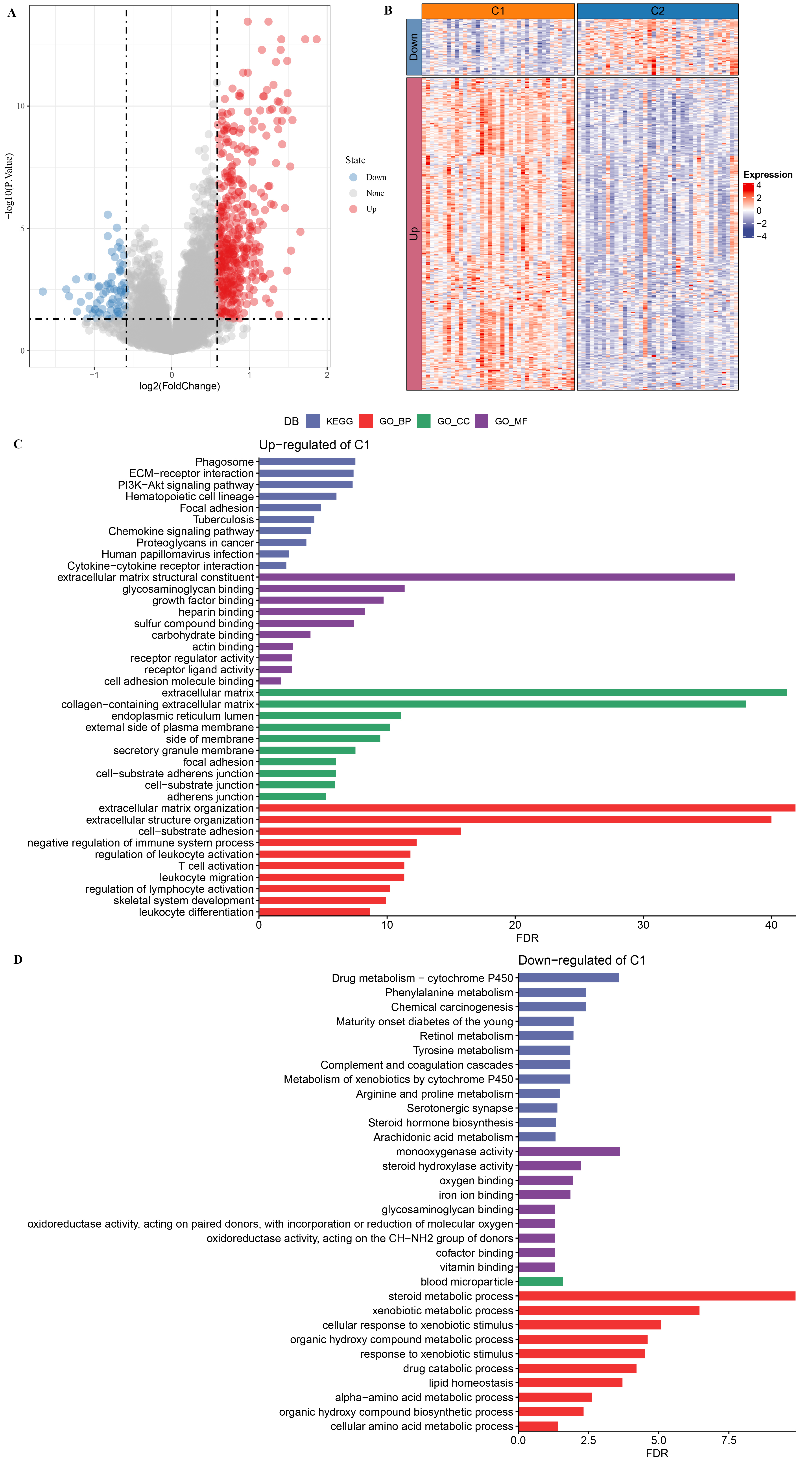

Supplement: Supplementary file 2 [file Image3.JPEG]

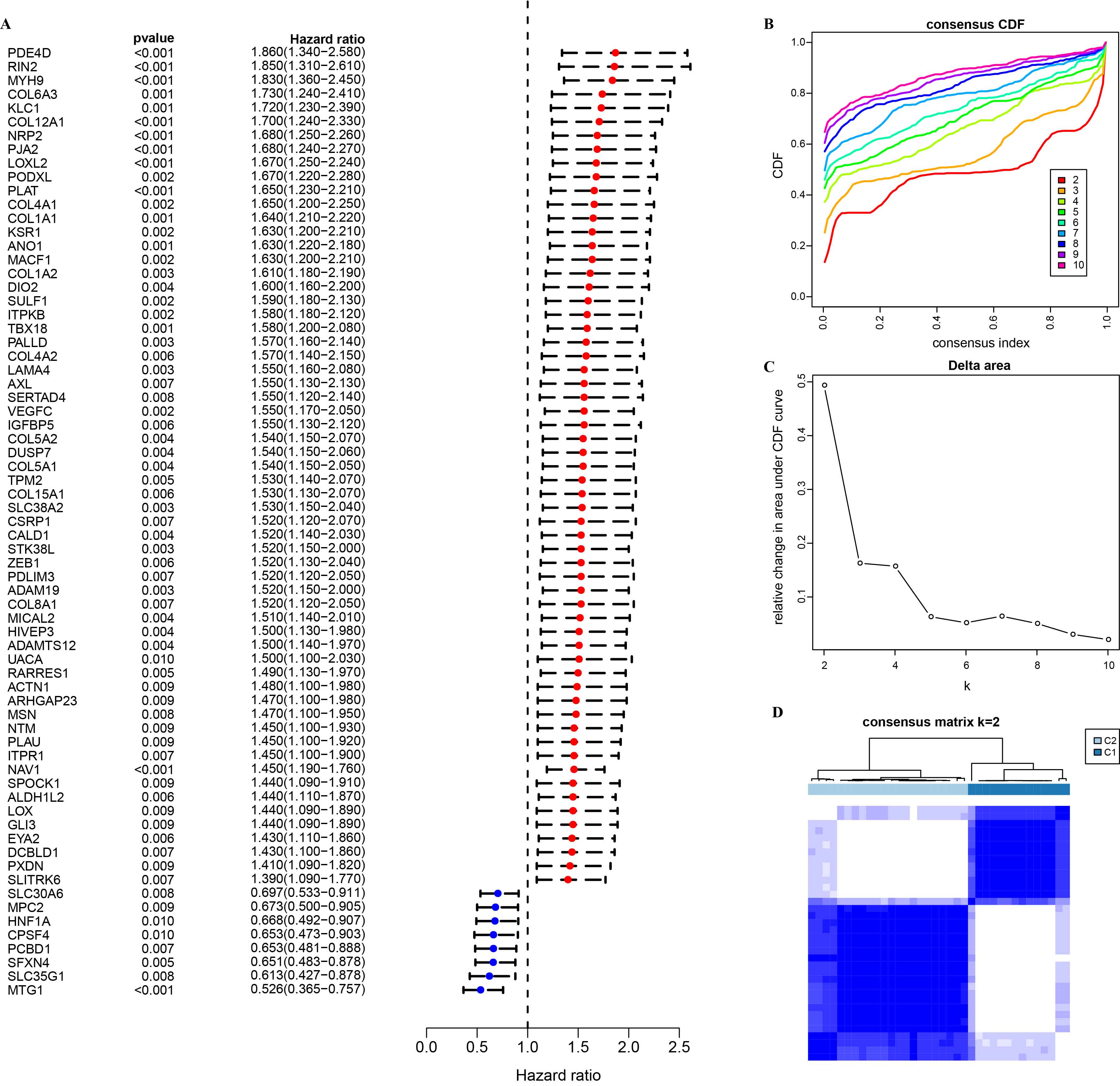

Supplement: Supplementary file 3 [file Image1.JPEG]

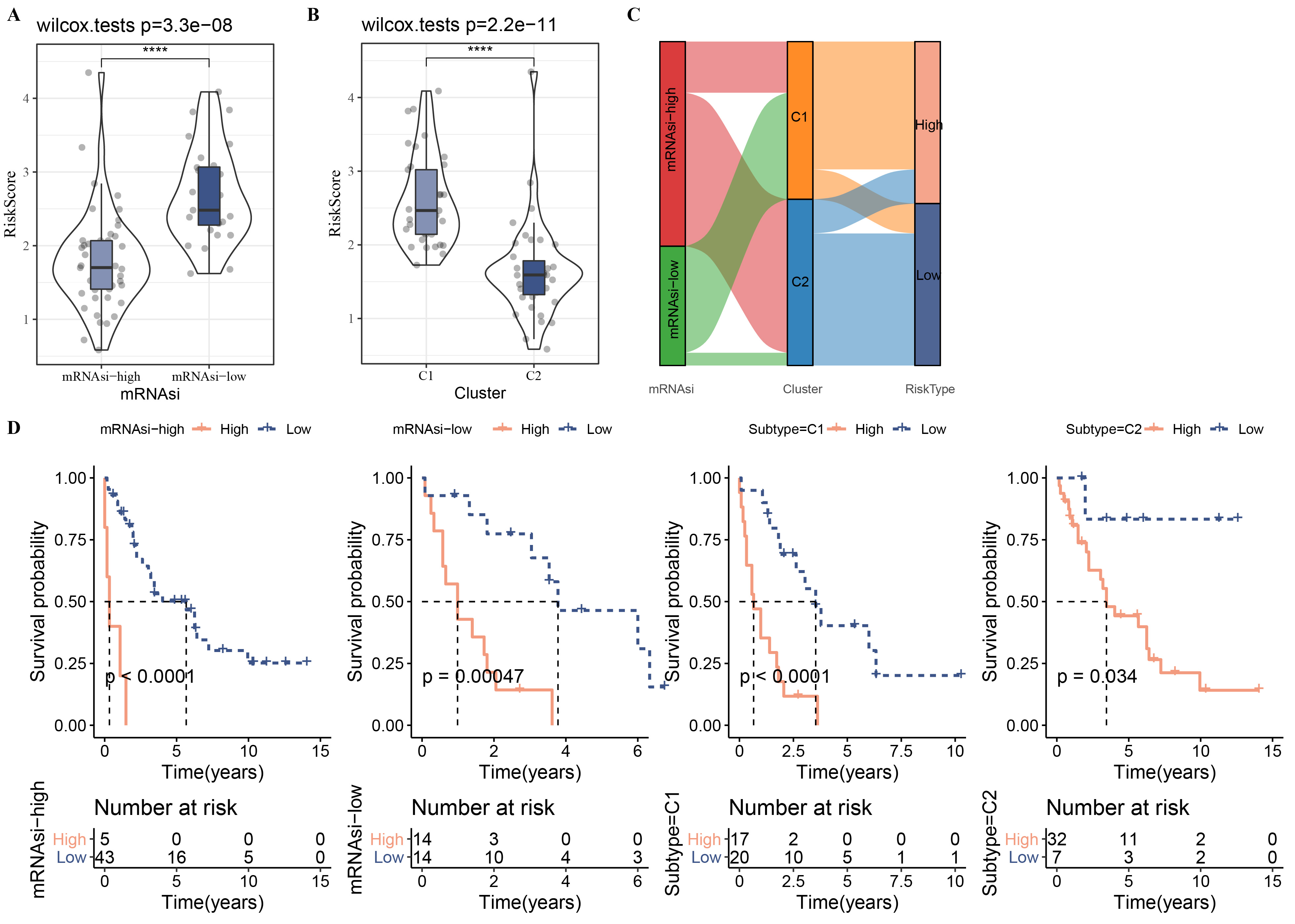

Supplement: Supplementary file 4 [file Image4.JPEG]

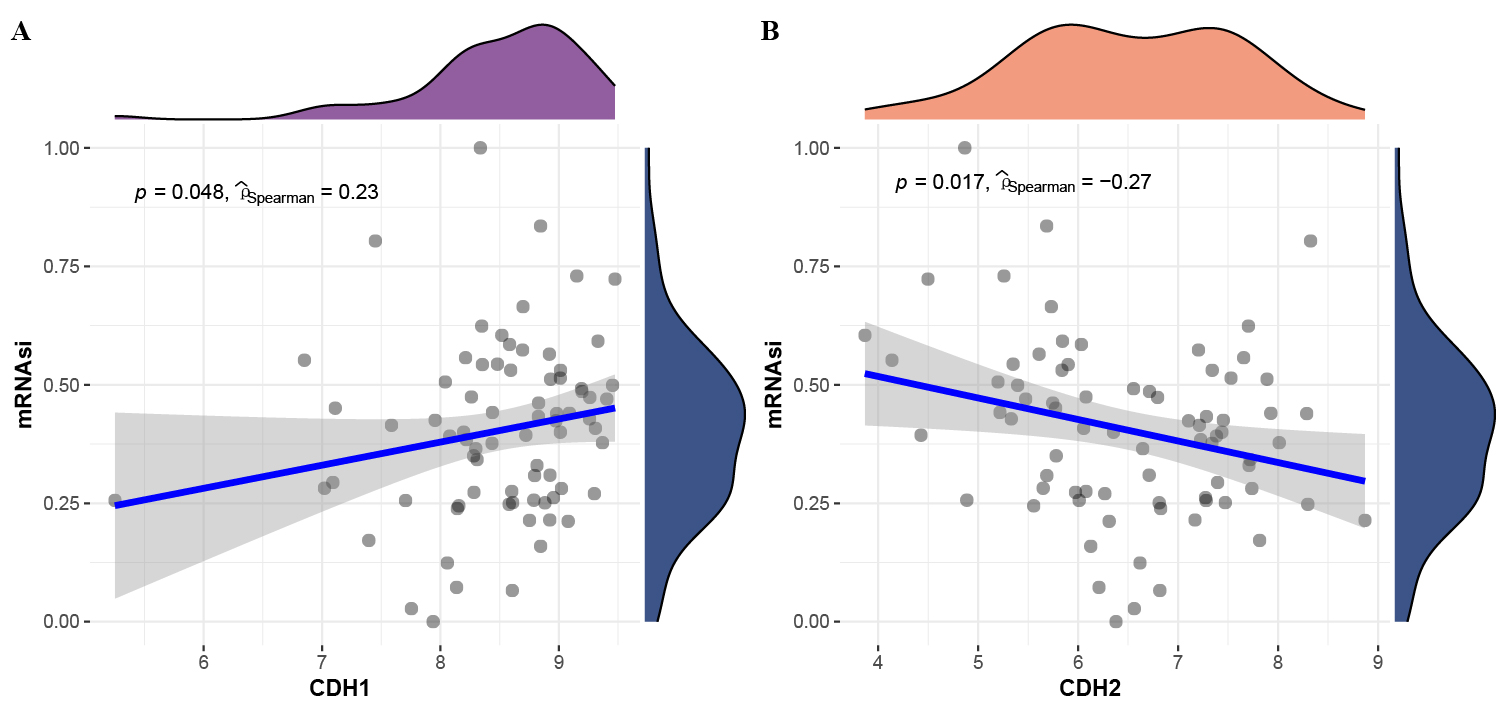

Supplement: Supplementary file 5 [file Image2.JPEG]

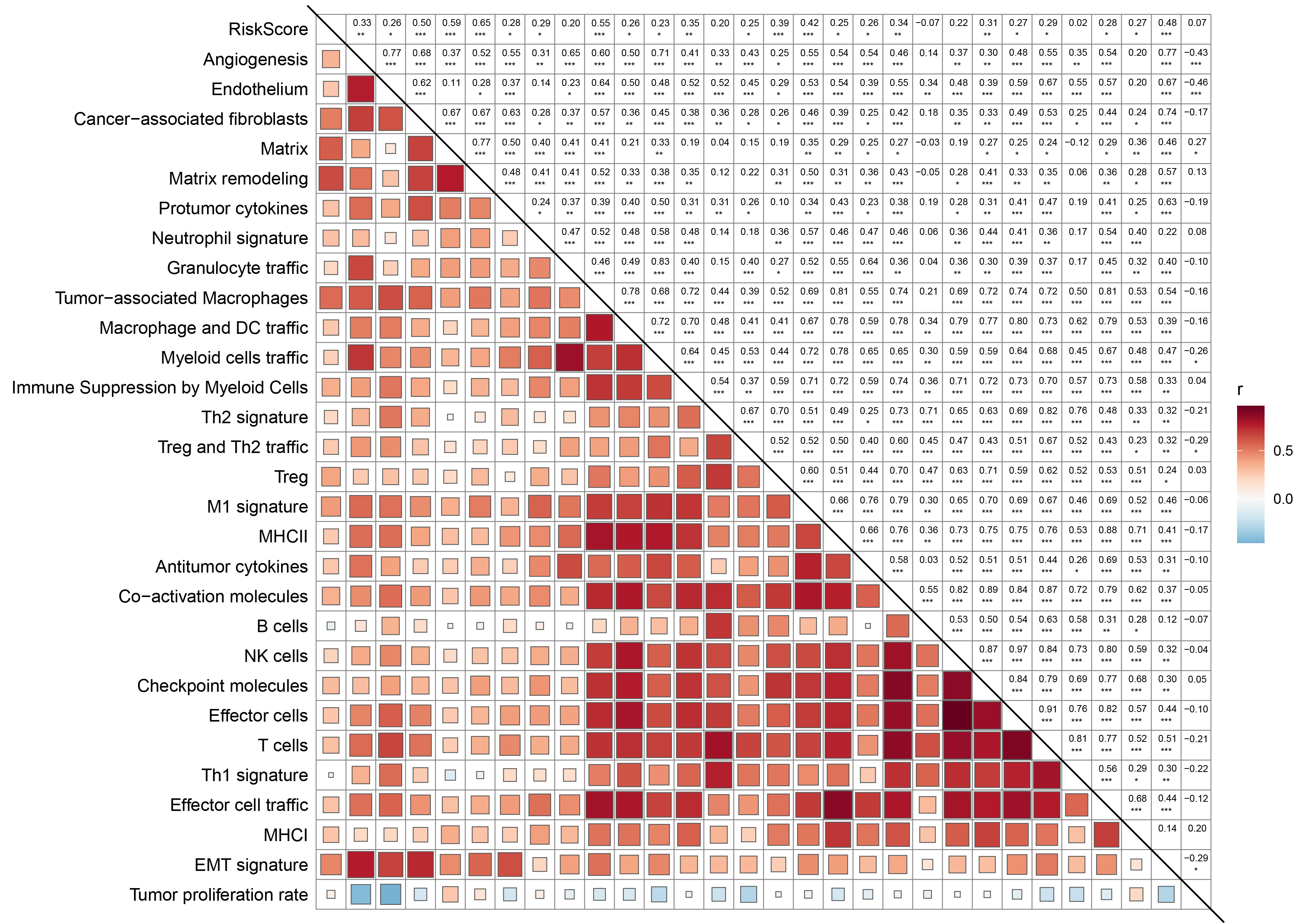

Supplement: Supplementary file 6 [file Image5.JPEG]
