# Supplementary material for: Exploring the role of tumor stemness and the potential of stemness-related risk model in the prognosis of intrahepatic cholangiocarcinoma
Source: Front Genet. 2023 Jan 12;13:1089405. doi: 10.3389/fgene.2022.1089405 (PMC9877308; doi:10.3389/fgene.2022.1089405)
Supplement: Supplementary file 7 [file Table3.DOCX]

Data analyzed in this study:

https://www.jianguoyun.com/p/Dci4IF0Q2fqNCxi_1OMEIAA
