# Supplementary material for: Exploring the role of tumor stemness and the potential of stemness-related risk model in the prognosis of intrahepatic cholangiocarcinoma
Source: Front Genet. 2023 Jan 12;13:1089405. doi: 10.3389/fgene.2022.1089405 (PMC9877308; doi:10.3389/fgene.2022.1089405)
Supplement: Supplementary file 9 [file Table4.DOCX]

Supplementary materials

Figure S1. Identification of molecular subtypes related to mRNAsi in E-MTAB-6389 cohort. (A) A total of 69 prognostic genes identified by univariate Cox regression. (B-C) Consensus CDF and area under CDF curve when cluster number k = 2-10. (D) Consensus matrix when k =2.

Figure S2. Spearman correlation analysis of mRNAsi with CDH1 and CDH2.

Figure S3. Identification of DEGs between C1 and C2. (A) Volcano plot of 473 DEGs. Red and blue represent up-regulated and down-regulated genes. (B) Heatmap of the expression of 473 DEGs in C1 and C2. (C-D) GO and KEGG analysis of up-regulated genes (C) and down-regulated genes (D). The top 10 enriched terms or pathways were displayed if the items were over than 10.

Figure S4. (A) Violin plot of risk score in mRNAsi-high and mRNAsi-low groups. (B) Violin plot of risk score in C1 and C2. (C) The distribution of two risk groups in subtypes and mRNAsi groups.

Figure S5. The correlation of risk score with immune-related characteristics.

Table S1. A list of 1746 DEGs with FDR < 0.05 and log2FC > 0.

Table S2. A list of 98 prognostic genes identified by unvariate Cox regression.
